# Neutrophil cell surface receptor dynamics following trauma: a systematic review

**DOI:** 10.1007/s00068-025-02937-0

**Published:** 2025-08-11

**Authors:** Christian T. Hübner, Lukas Schulte, Felix Klingebiel, Emma de Fraiture, Yannik Kalbas, Alba Shehu, Falco Hietbrink, Roman Pfeifer, Hans-Christoph Pape, Michel P. J. Teuben

**Affiliations:** 1 Department of Traumatology, University Hospital Zurich, University of Zurich, Raemistrasse 100, 8091 Zurich, Switzerland; 2 Department of Trauma Surgery, University Medical Center Utrecht, Utrecht, Netherlands

**Keywords:** Neutrophils, Systematic review, Trauma, Cell surface markers, Immunomonitoring, Neutrophil activation, Polytrauma, Post-traumatic immune response

## Abstract

**Purpose:**

Neutrophils are essential effector cells in the immune response to traumatic injury. Although changes in receptor expression over time have been described in the literature, effective monitoring strategies are still lacking. This systematic review aims to identify reported neutrophil cell surface receptor dynamics after trauma and to determine the post-traumatic neutrophil signature over time, forming the basis for future immunomonitoring.

**Methods:**

We conducted a systematic review to identify clinical studies describing neutrophil cell surface expression levels of relevant markers in the field of trauma. Reported post-traumatic alterations were gathered and pooled.

**Results:**

A total of 1,266 publications were identified that used neutrophil phenotype in a clinical setting, of whom 34 were utilized for data extraction. The most frequently analyzed receptors were Mac-1/CD11b (*n* = 25), L-selectin/CD62L (*n* = 9). Fifteen studies investigated the *Fcγ-*receptor family. CD11b increases after moderate to severe trauma, peaking at 24 h, then decreases over 6 months. L-selectin shows early fluctuations, whereas CD16 decreases homogeneously after trauma. CD64, as well as other less well-described markers (CD13, CD46, CD55, CD59) show homogenous alterations following injury.

**Conclusions:**

This study is the first comprehensive review on post-traumatic neutrophil receptor dynamics. By pooling available data from literature, a unique multi-parameter post-traumatic neutrophil signature, with both traditional neutrophil markers (CD11b, CD11a, CD62L, CD16, CD64) and novel markers has been identified. Future combining of these markers and subset analysis may form the basis for post-traumatic immunomonitoring.

**Supplementary Information:**

The online version contains supplementary material available at 10.1007/s00068-025-02937-0.

## Introduction

Trauma activates the immune system. Inappropriate activation of the immune system forms the basis for life-threatening inflammatory complications such as acute respiratory distress syndrome (ARDS), sepsis and multiple organ dysfunction syndrome (MODS) [1, 2]. Patients with multiple injuries (i.e. polytrauma patients), are at increased risk of complicated post-traumatic courses [3].

Due to evolution in treatment strategies, polytrauma patients showed a decreasing trend in mortality over the last decades. This is mostly due to improved pre- and in-hospital trauma care, triage on a foundation of better understanding of the pathophysiology of polytrauma [4, 5]. While many of the deaths occur “early” in the first 24 h, mostly as result of exsanguinating hemorrhage, head injury and overactivation of the immune system [6], “late” trauma fatalities are believed to be caused by a dysfunctional immune response [7, 8].

The complexity of the immune response following trauma is not entirely understood, however neutrophils seem to play an essential role. Over- und underperforming of neutrophils after trauma forms the basis for the development of ARDS, sepsis and MODS. Monitoring of systemic neutrophils, is therefore promising to predict outcome and to guide timing and intensity of surgical intervention (e.g. fracture fixation: early total care, primary fixation/damage control surgery, or even early appropriate care) [9].

PNM dynamics after trauma have already been investigated in both animal and human studies [10–13]. Recent studies could demonstrate the feasibility of fully automated point-of-care (POC) flow-cytometry analysis of PNM dynamics in trauma patients [14–16]. Currently, literature on neutrophil activation after trauma is scarce and scattered, due to difficulty in analysis, heterogeneity in patient cohorts, trauma severity, treatment, and differences in sampling as well as laboratory protocols. This neutrophil activation analysis might be a viable option for immunomonitoring to guide prevention, treatment and long term prognosis. Nevertheless, in order to identify relevant neutrophil markers for future immunomonitoring of traumatized patients, it is key to gain an overview of available data from existing clinical trauma studies. This allows for data pooling, identification of novel markers/subtypes and is a prerequisite for the development of future multifactorial automatized (POC)-neutrophil studies. Therefore, the aim of the current review is to identify reported cell surface receptor expression alterations after insult from clinical trauma studies [17].

## Material & methods

The reporting of this systematic review was conducted in accordance with the Preferred Reporting Items for Systematic Reviews and Meta-Analyses (PRISMA) guidelines (http://www.prisma-statement.org/).

### Eligibility criteria

A systematic review was conducted to identify all relevant original clinical publications reporting neutrophil receptor expressions after injury. Only original articles written in English or German were included. Exclusion criteria included studies on pediatric patients, experimental studies, animal trials, case reports, case series, reviews, editorials, low-quality studies.

### Information sources and search strategy

The Medline and EMBASE databases were searched to cover the period from January 1, 1995, to May 10, 2024. The search strategy included a combination of controlled vocabulary terms (MeSH/Emtree terms) and regular search terms, connected using Boolean operators. Truncation was applied to account for plural forms and alternate spellings, and careful attention was paid to include all relevant synonyms. Filters were applied to exclude inappropriate article types. The full list of search terms is provided in Supplementary File 1. The search results were organized and deduplicated using Clarivate™ EndNote™ version 20.

### Study selection

Titles and abstracts of the identified articles were independently screened by LS, FKLK, CTH and MT. CTH and MT cross-checked the extracted data, and any disagreements were resolved through personal discussion. Full texts of potentially eligible studies were retrieved through the university’s central library via respective publishers.

### Data extraction

Reported information regarding any alteration of neutrophil receptor activation after trauma was extracted from the included articles. Information for each reported receptor was grouped and analyzed in a qualitative approach. Trends for respective alterations (*no/increased-/decreased-activation*) was grouped for each reported receptor and visualized. Furthermore, time-windows and study characteristics have been reported. Additionally, characteristics of the studies, patients and trauma was extracted and outlined.

## Results

**Fig. 1:**
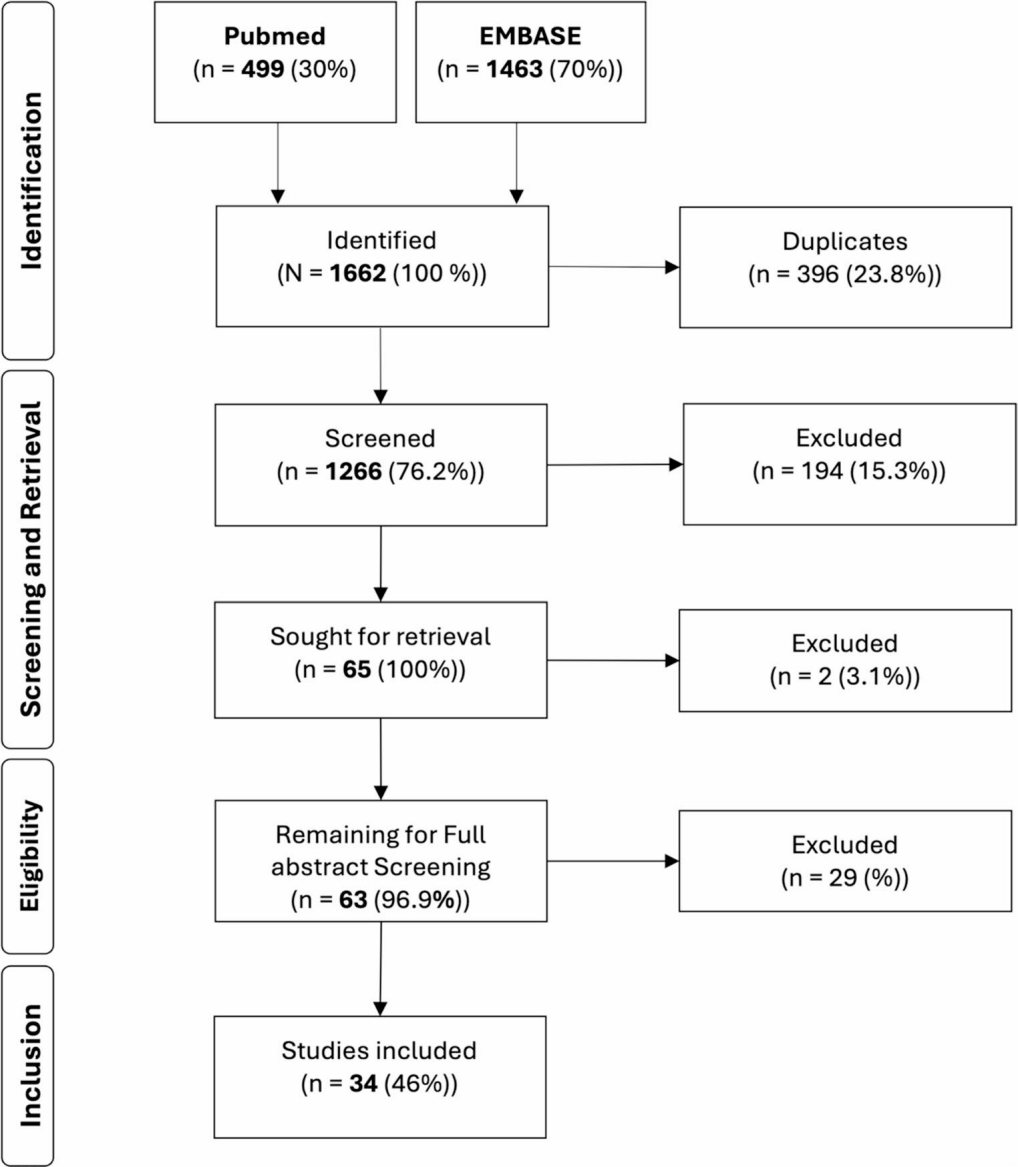
Flowchart of the screening process

A total of 1,266 publications were identified and screened for eligibility. Finally, 34 articles reporting on clinical studies with neutrophil receptor expression analysis, were included. Figure 1 provides a flowchart on study selection. An overview of selected studies for data-extraction has been shown in Tables 1 and 2.

**Table 1:** Study overview and clinical parameters of identified studies

Author	Title	Country	Year	Journal	Included markers	Patients	Age (Mean)	ISS (Median)
Spijkerman et al.	Point-of-Care Analysis of Neutrophil Phenotypes: A First Step Toward Immuno-Based Precision Medicine in the Trauma ICU	Netherlands	2020	Critical Care Explorations	CD16, CD62l, CD35, CD11c, CD11b, CBRM1/5, CD10, and CD66b	32	n.a.	n.a.
Nijdam et al.	Identification of neutrophil phenotype categories in geriatric hip fracture patients aids in personalized medicine	Netherlands	2024	OTA International	CD10, CD11b, CD16, CD62L, CD64	52	82,0	n.a.
Botha	Base deficit after major trauma directly relates to neutrophil CD11 b expression: a proposed mechanism of shock-induced organ injury	United States	1997	Intensive Care Medicine	CD11a, CD11b, CD18	17	26,7	26.7 (mean)
Maekawa et al.	Effects of trauma and sepsis on soluble L-selectin and cell surface expression of L-selectin and CD11b	Japan	1998	The Journal of Trauma: Injury, Infection, and Critical Care	CD11b, CD62l	35	42,3	13.8 (mean)
Johansson et al.	Dynamics of leukocyte receptors after severe burns: An exploratory study.	Sweden	2011	Burns	CD11b, CD14, CD16, CD62l	10	29,6	n.a.
Groeneveld et al.	Early decreased neutrophil responsiveness is related to late onset sepsis in multitrauma patients: An international cohort study	Netherlands	2017	PLoS One	CD11b, CD32	109	35,6	26 (mean)
Hazeldine et al.	Prehospital immune responses and development of multiple organ dysfunction syndrome following traumatic injury: A prospective cohort study.	United Kingdom	2017	PLoS Med	CD11b, CD16, CD62L, CD63, CD88, CD181, CD182	89	41,0	24 (mean)
Bhatia et al.	Modulation of Interleukin-8-Mediated Neutrophil Migration Following Major Lower-Limb Fracture and Operative Stabilization.	United Kingdom	2005	European Journal of Trauma	CD11b, CD18, CD31, CD181	18	37,4	n.a.
Hietbrink et al.	Kinetics of the innate immune response after trauma: implications for the development of late onset sepsis.	Netherlands	2013	Shock	CD11b, CD32, CD88, CD181	36	45 (median)	24
Shih et al.	Alternations of surface antigens on leukocytes after severe injury: correlation with infectious complications.	Taiwan	1998	Intensive Care Med	CD11b, CD16, CD25	16	41,4	24.7 (mean)
Weiss et al.	Polymorphonuclear cell surface expression patterns differ in inflammatory and infectious stages in polytraumatized and septic shock patients.	Germany	2014	Critical Care	CD11b, CD16, CD88, CD64, CD66b, CD95, CD33, CD39,	8	n.a.	n.a.
Kasten et al.	Divergent adaptive and innate immunological responses are observed in humans following blunt trauma.	United States	2010	BMC Immunology	CD11b	22	36,3	22.8 (mean)
Bhatia et al.	Enhanced neutrophil migratory activity following major blunt trauma.	United Kingdom	2005	Injury	CD11b, CD18, CD31, CD181	11	36,6	22
Visser et al.	Isolated blunt chest injury leads to transient activation of circulating neutrophils	Netherlands	2011	Eur J Trauma Emerg Surg	CD11b, CD32, CD64, CD62l, CD88, CD181, CD182	11	54,4	17
Agudelo et al.	Influence of preoperative 7.5% hypertonic saline on neutrophil activation after reamed intramedullary nailing of femur shaft fractures: a prospective randomized pilot study	United States	2012	J Orthop Trauma	CD11b, CD62l, CD66b	20	43,5	11 (mean)
Baëhl et al.	Altered neutrophil functions in elderly patients during a 6-month follow-up period after a hip fracture	Canada	2015	Exp Gerontol	CD11b, CD16, CD32, CD88, CD66b, CD282, CD284	34	75,9	n.a.
Scannell et al.	Effects of trauma on leukocyte intercellular adhesion molecule-1, CD11b, and CD18 expressions	United States	1995	J Trauma	CD11b, CD18, CD54	10	33,0	n.a.
Brom et al.	Expression of the adhesion molecule CD11b and polymerization of actin by polymorphonuclear granulocytes of patients endangered by sepsis	Germany	1995	Burns	CD11b	16	42,0	n.a.
Bhatia et al.	Neutrophil priming for elastase release in adult blunt trauma patients	United Kingdom	2006	J Trauma	CD11b, CD18	23	n.a.	n.a.
Briggs et al.	Biomarkers to Guide the Timing of Surgery: Neutrophil and Monocyte L-Selectin Predict Postoperative Sepsis in Orthopaedic Trauma Patients	Australia	2021	J Clin Med	CD11b, CD18, CD62l, CD64, CD181	162	44,2	n.a.
Hietbrink et al.	Modulation of the innate immune response after trauma visualised by a change in functional PMN phenotype	Netherlands	2009	Injury	CD11b, CD32	52	34 (median)	10
Hietbrink et al.	The Impact of Intramedullary Nailing of Tibia Fractures on the Innate Immune System	Netherlands	2015	Shock	CD11b, CD32	25	42,5	9.4 (mean)
Hietbrink et al.	Intramedullary nailing of the femur and the systemic activation of monocytes and neutrophils	Netherlands	2011	World Journal of Emergency Surgery	CD11b, CD32	38	30 (median)	13
Hietbrink et al.	Aberrant regulation of polymorphonuclear phagocyte responsiveness in multitrauma patients	Netherlands	2006	Shock	CD11b, CD18	13	45,7	21
Huschak et al.	Changes in monocytic expression of aminopeptidase N/CD13 after major trauma	Germany	2003	Clin Exp Immunol	CD13	30	39,0	31 (mean)
Groeneveld et al.	Early Dysfunction Of FcgammaRII (CD32) On Neutrophils Preceeds Late Onset Septic Complications In Multi Trauma Patients, in A24.	Netherlands	2010	American Thoracic Society.	CD16, CD32	n.a.	n.a.	n.a.
Amara et al.	Early expression changes of complement regulatory proteins and C5A receptor (CD88) on leukocytes after multiple injury in humans	Germany	2010	Shock	CD35, CD46, CD55, CD59, CD88	12	38,6	n.a.
Mommsen et al.	Regulation of L-selectin expression by trauma-relevant cytokines	Germany	2011	Pathol Res Pract	CD62l	20	33,1	n.a.
Cocks et al.	Leukocyte L-Selectin Is Up-Regulated after Mechanical Trauma in Adults	Hong Kong	1998	Journal of Trauma and Acute Care Surgery	CD62l	41	41 (median)	10
Raikwar et al.	Analysis of Risk Factors and Association of Cluster of Differentiation (CD) Markers With Conventional Markers in Delayed Fracture Related Infection for Closed Fracture	India	2021	Cureus	CD64, CD66b	510	47,8	n.a.
Wang et al.	[Dynamic changes of cellular immune function in trauma patients and its relationship with prognosis]	China	2021	Zhonghua Wei Zhong Bing Ji Jiu Yi Xue	CD64, CD279	42	n.a.	n.a.
Yuanyuan et al.	Serum PCT, CRP and CD64 levels in predicting early infection after internal fixation of limb fractures	China	2023	Chinese Journal of Clinical Infectious Diseases	CD64	2572	n.a.	n.a.
Tarlowe et al.	Prospective study of neutrophil chemokine responses in trauma patients at risk for pneumonia	USA	2005	Am J Respir Crit Care Med	CD181, CD182	32	35,1	27.4 (mean)
Quaid et al.	Preferential Loss of CXCR-2 Receptor Expression and Function in Patients Who Have Undergone Trauma	USA	1999	Archives of Surgery	CD181, CD182	20	35,0	n.a.

**Table 2:** Timepoints of measurements for each neutrophil surface receptor

Antibody	Timepoint	Upregulation	Downregulation	Unaltered
CD10	1 h	1		1
CD11a	24 h		1	
CD11b	1 h	6	2	7
	3 h	1		
	6 h	1	1	
	9 h		1	
	24 h	4	1	
	48 h	1		
	72 h	1	2	
	96 h	1		
	120 h	3		
	144 h	1		
	168 h	2	1	
	240 h	1		
	6 weeks	1		
	6 months			1
CD11c	1 h			1
CD13	1 h		1	
	14d		1	
CD16	1 h	1	2	
	3 h		1	
	4 h		1	
	9 h		1	
	24 h		3	
	48 h		2	
	72 h		1	
	96 h		1	
	120 h		1	
	144 h		1	
	168 h		2	
	6 weeks			1
	6 months			1
CD18	1 h			4
	24 h			1
	72 h		1	
	120 h			1
CD181	1 h	1		
	24 h	1		
	72 h	1		
	120 h	1		
CD182b	1 h		3	
	3 h		1	
	4 h		1	
	9 h		1	
	24 h		1	
	72 h		1	
CD31	1 h		1	
	24 h		1	
	168 h			1
CD32	1 h		3	3
	3 h		1	
	9 h		1	
	24 h		1	
CD46	4 h		1	
	12 h		1	
	24 h		1	
	48 h		1	
	120 h		1	
	240 h		1	
CD55	1 h			1
	4 h			0
	12 h			0
	24 h		1	
	48 h		1	
	120 h		1	
	240 h		1	
CD59	1 h	1		
	4 h	1		
	12 h	1		
	24 h	1		
	48 h	1		
	120 h	1		
	240 h	1		
CD62L	1 h	1	2	2
	3 h	1		1
	7 h		1	
	9 h		1	
	24 h		2	1
	48 h	1	0	
	72 h		1	
CD63	1 h			1
	4 h			1
	72 h			1
CD64	1 h	4		
	7 h	1		
	24 h	1		
	72 h	2		
	168 h	1	1	
CD66b	1 h	1		1
	72 h	1		
	168 h	1		
CD88	1 h		2	
	3 h		1	
	9 h		1	
	24 h		2	
	48 h		1	
	72 h		1	
	168 h		1	
	6 weeks		1	
	6 months			1

### Frequency of reported cell surface receptors in trauma literature

**Fig. 2:**
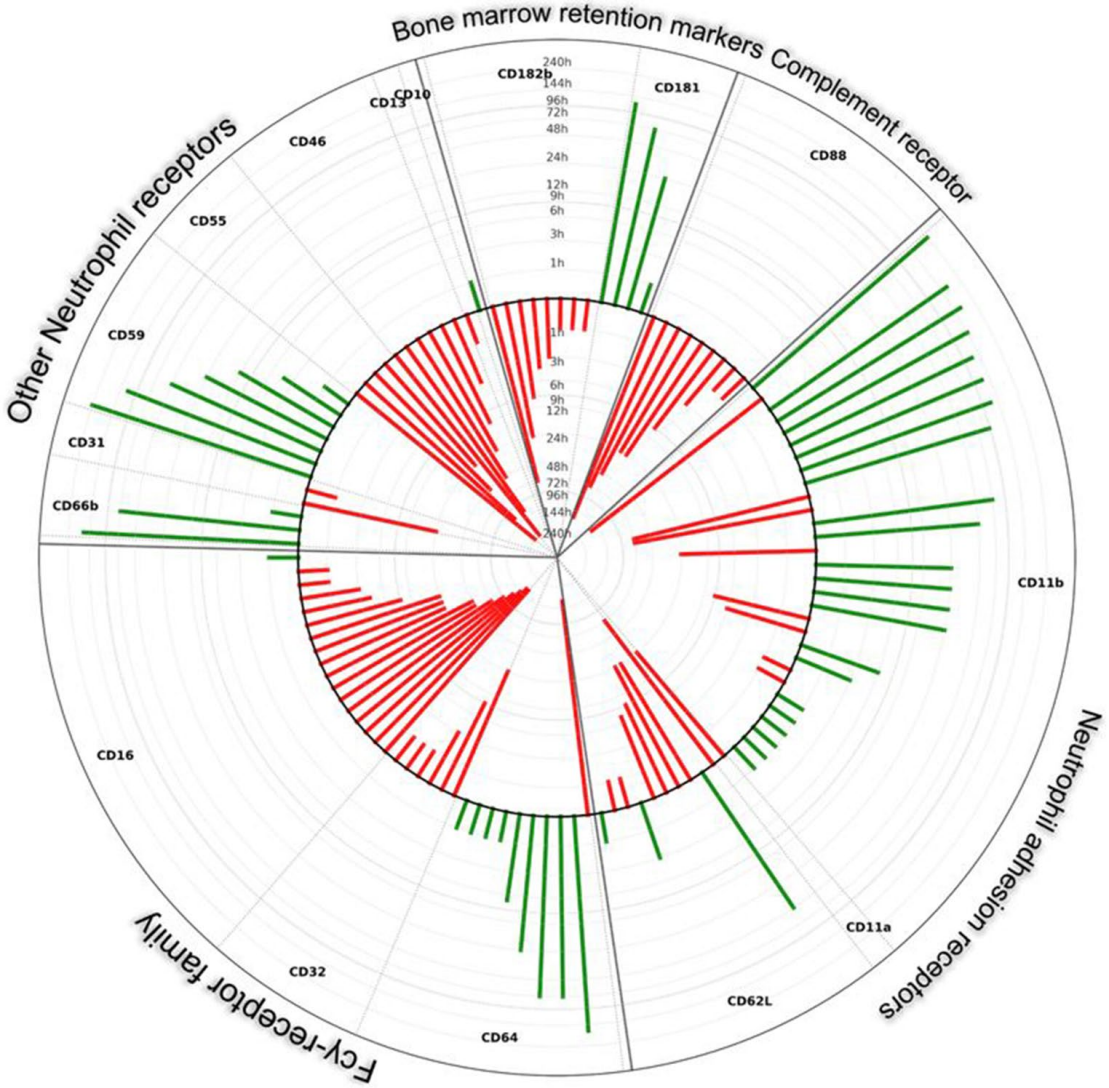
Overview on expression patterns after trauma. Green columns in direction to outside show over expression after trauma. Red columns to center show under expression. Height of the columns reflects timepoint of measurement

The most frequently analyzed parameter in trauma literature was the integrin receptor Mac-1/CD11b (25 studies), followed by L-selectin/CD62L (9 studies). Fifteen studies investigated post-traumatic changes in the *Fcγ*-receptor family. Hereafter, an overview of specific neutrophil receptor-clusters and observed effect for specific receptors upon trauma have been provided (Fig. 2).

### Alterations in neutrophil ***Fcγ***-receptor family receptors

*Fcγ* receptors (*FcγRs*) play a crucial role in neutrophil-mediated immune responses by binding to the Fc region of immunoglobulin G (IgG), facilitating phagocytosis, antibody-dependent cellular cytotoxicity (ADCC) and inflammatory signaling. In neutrophils three main classes of *FcγRs* are expressed: CD16 (*FcγRIII*), CD32 (*FcγRII*), and CD64 (*FcγRI*). Each of them have different affinities and functions:

1. *FcγRIII (CD16)*:
  CD16 is a low-affinity Fc receptor expressed on a subset of neutrophils. Six studies could be identified, investigating changes in CD16 after trauma. CD16 decreased in all studies homogeneously, especially in the first 24 h after trauma [18–23].
2. *FcγRII (CD32)*:
  Seven studies investigated alterations after trauma and orthopedic operations. In four studies a decreasing compared to control were obvious [20, 24–26], while in three studies no significant alterations could be detected [21, 27, 28].
3. *FcγRI (CD64)*:
  CD 64 is a high-affinity Fc receptor. Four studies could be detected investigating regulation after trauma. In all studies, expression was higher than healthy controls in all clinical groups after trauma, especially after severe trauma and surgery [13, 29–31].

### Alterations in neutrophil adhesion receptors (selectins and integrins)

Adhesion receptors are essential for their recruitment, migration, and interaction with the endothelium during immune responses. These receptors can be categorized into two major groups (selectins (CD62L) and integrins (CD11a, CD11b, CD11c).

#### L-selectin (CD62L)

L-Selectin is a key adhesion molecule that mediates their initial tethering and rolling on the vascular endothelium. It binds to glycoproteins such as peripheral node addressins (PNAd) and mucosal vascular addressin cell adhesion molecules (MAdCAM-1) on endothelial cells. Shedding of CD62L upon neutrophil activation facilitates their transition from rolling to firm adhesion and transmigration [32]. Nine studies could be identified, investigating alterations in L-selectin after trauma and operations. In six studies a decrease of cell surface expression of CD62L after trauma could be detected [13, 20, 22, 33–35]. In two studies no significant expression levels could be detected [18, 36] and in one study an early increase after trauma was evident [37].

#### Integrins (CD11a, CD11b, CD11c)

Integrins are heterodimeric receptors that mediate firm adhesion and transendothelial migration. CD11a (LFA-1) and CD11b (Mac-1), in association with CD18, bind to intercellular adhesion molecule-1 (ICAM-1) and ICAM-2, stabilizing neutrophil adhesion to the endothelium. CD11c (αXβ2) is involved in immune complex recognition and phagocytosis [38]. Mac-1/CD11b was the most investigated parameter over all (25 studies). In most of the studies (14 studies) CD11b expression was significant higher after trauma, resulting in an activation of the neutrophils [18, 19, 22–26, 33, 35, 39]. In these studies, it increases after moderate to severe trauma, peaking at 24 h, then decreases, with prolonged elevation in severe cases up to 6 weeks, returning to homeostatic levels by 6 months. However, six studies reported lower CD11b expression after trauma [20, 21, 34, 40–42]. In five studies no significant alterations after trauma could be detected [13, 16, 27, 28, 43]. One study each investigated changes in CD11a and CD11c after trauma. In CD11a lower levels could be detected after trauma [44], while in CD11c no significant changes were evident [16].

### Alterations in neutrophil-complement interactions

#### Complement C5aR (CD88)

CD88 is the primary receptor for complement component C5a and plays a crucial role in neutrophil chemotaxis and activation. Six studies investigated changes in this receptor after trauma. In all studies CD88 was decreased in a time range from immediately after trauma up to 7 days posttraumatic [20–23, 45].

#### Complement receptor type 1 (CD35)

CD 35 is a membrane protein on neutrophils that mediates the clearance of immune complexes by binding to complement components. It also regulates complement activation, influences neutrophil adhesion, and plays a role in immune modulation. Two studies investigated alterations after polytrauma. One study revealed early depression compared to healthy volunteers and a recovery over 48 h post polytrauma [45], while another showed no signs of direct activation in monotrauma or polytrauma patients. No significant differences between polytrauma patients with or without infectious complications during hospital admission were evident [16].

### Chemokine receptors and neutrophil bone marrow retraction markers

Chemokine receptors CD181 (CXCR1) and CD182b (CXCR2) are essential in neutrophil migration and activation by binding to CXC chemokines, such as IL-8 (CXCL8). CD181 mediates neutrophil activation and oxidative burst, while CD182 is more involved in chemotaxis and neutrophil recruitment to sites of inflammation.

#### CXCR1 (CD181)

Seven studies investigated the regulation of CD181 after trauma. The studies present conflicting findings regarding the expression. Three studies report an early rise after trauma, lasting up to five days or already elevated at admission [20, 40, 43]. However, in three studies the levels decreased after trauma [13, 22, 24] and in one study now relevant changes could be detected [46]

#### CXCR2 (CD182)

In four studies a homogenously decrease of expression after trauma was detected. These changes are reported immediately after trauma, as well as after four to 72 h [20, 22, 46, 47].

### Other neutrophil receptors with posttraumatic alterations

#### CD13 (aminopeptidase N)

CD13 is a membrane-bound enzyme, which plays a role in immune regulation and inflammatory responses, especially in oncogenesis. Its expression influences neutrophil function and migration. Only one study was identified revealing changes in neutrophil expression after trauma. Immediately after trauma, expression was lower than in healthy controls, slowly increasing to normal levels over 14 days [48].

#### CD46 (membrane cofactor protein)

CD46 is a regulatory protein expressed on neutrophils that plays a key role in controlling complement activation and preventing excessive immune responses. It also influences neutrophil survival, activation and cytokine production during inflammation. A decrease 1 h post polytrauma with lowest levels of neutrophil cell surface expression at 24 h posttraumatic have been described in one included study. After 10 days no changes in expression levels in comparison of healthy volunteer could be measured [45].

#### CD55 (decay-accelerating factor, DAF)

CD 55 is a complement regulatory protein expressed on neutrophils that protects cells from complement-mediated damage by inhibiting C3 and C5 convertases. It plays a role in modulating immune responses and can be upregulated during inflammation. One included study investigated this factor after major trauma. CD55 increased 24 h post polytrauma with a peak at 48 h and decreased after 120 h to normal levels [45].

#### CD63 (lysosome-associated membrane glycopreotein 3, LAMP3)

CD63 is a tetraspanin membrane protein that plays a role in degranulation and cell activation in neutrophils. It is involved in regulating inflammatory processes and is often used as a marker for activation and vesicle exocytosis. One study in this review showed no statistically significant changes after polytrauma [22].

#### CD59 (protectin)

CD59 as a complement regulatory protein on neutrophils inhibits the formation of the membrane attack complex (MAC), preventing complement-mediated cell lysis. Furthermore, it contributes to immune regulation and protects cells from excessive inflammatory damage.

In one study increased levels early after admission in polytrauma patients who developed sepsis could be measured [45].

#### CD10 (membrane metallo-endopeptidase (MME), neutral endopeptidase (NEP), common acute lymphoblastic leukemia antigen (CALLA)

CD10 is considered as a stratification marker for maturation state of circulatory neutrophils both in chronic and acute systemic inflammatory conditions Loss of CD10 expression is associated with immature and dysfunctional neutrophils, which can contribute to inflammatory disorders and impaired immune regulation [49, 50]. One study revealed higher expression levels after trauma [35]. A second study however, did not show statistic significant changes between trauma and control group [16].

#### CD31 (Platelet/endothelial cell adhesion molecule-1, PECAM-1)

CD31 is the platelet endothelial cell adhesion molecule-1 (PECAM-1). This transmembrane glycoprotein is expressed on neutrophils, endothelial cells and platelets and is involved in cell migration, angiogenesis and cell survival. In neutrophils it plays an essential role in cell-cell adhesion. Two studies on trauma could be identified. In both studies, CD31 was significant downregulated after trauma compared to healthy controls [40, 51].

#### CD66b (Carcinoembryonic antigen-related cell adhesion molecule 8, CEACAM8)

CD66b is a granulocyte-specific surface marker predominantly expressed on neutrophils. Its functionality is diverse and CD66b plays a role in cell adhesion and activationUpon neutrophil activation, CD66b expression increases, facilitating interactions with endothelial cells and enhancing neutrophil degranulation, phagocytosis, and cytokine release. Its upregulation is often associated with inflammatory responses and infections [52]. Two studies could identify higher expression levels after trauma [23, 29], while one study did not show any differences between trauma and control group [16].

## Discussion

The current study is the first to provide a comprehensive overview of post-insult cell surface receptor expression alterations from clinical trauma studies. It was demonstrated that in addition to well-known neutrophil activation markers, including CD11b and CD62L, other less-well described, neutrophil markers also show homogenous post-traumatic cell-surface receptor dynamics. By pooling data from all existing literature on post-traumatic neutrophil changes the current study reveals a comprehensive post-traumatic systemic neutrophil signature. Combined analysis of both traditional and more exotic neutrophil markers after insult may allow for future immunomonitoring.

The introduction of automatized flow analysis enabled researchers to perform standardized, cheap and fast neutrophil studies from blood samples. Point-of-Care diagnostics of systemic neutrophil homeostasis in trauma bay or on the intensive care is a promising tool to monitor the trauma patient’s immune status and to predict outcome and the impact of additional (invasive) surgical interventions in the future. The feasibility of POC-diagnostics in trauma bay are promising, not only in the trauma setting but also on the ICU [14–16]. A prerequisite for the implementation of POC-neutrophil monitoring is the identification of markers that predict outcome best.

Currently, due to the availability of mainly heterogeneous clinical studies on trauma, data on post-insult neutrophil responses are scattered and findings differ markedly between studies. The current study is the first to combine all data from recent literature on clinical trauma studies. It became clear that not only traditional activation markers, but also (non-typical markers for activation) show post-traumatic alterations.

Essential for selecting markers for future immuno-monitoring are consistent effects/cell surface receptor expression dynamics upon post-traumatic activation. In fact, the known biological function of specific receptors should not be necessarily involved in post-traumatic activation. Post-traumatic neutrophil alterations due to bystander effects, with adequate and predictable cell surface receptor expression kinetics, may be suitable as marker for immuno-monitoring as well. Cell surface expression receptors that display heterogeneous effects, or in some studies no effect at all or inconsistent findings, are of less interest for early immuno-monitoring. Although, it may be, that these markers are of relevance for later studies on subgroups (personalized medicine).

As anticipated, integrin and selectin markers have been reported on most frequently in trauma literature. These markers are well-known and have been validated in both in vivo and in vitro studies. It has been demonstrated that L-selectin is shredded from the cell-membrane upon activation. Interestingly, this effect is only seen in 6/9 clinical trauma studies. An increase of L-selectin has also been described after trauma. Regarding integrins, only in 14/25 studies CD11b was upregulated after trauma. CD11a had lower after trauma [53], while in CD11c no significant changes were evident [16]. It is tempting to hypothesize that post-traumatic neutrophil activation entails a combination of both systemic cellular activation and de-activation. These processes may occur simultaneously, which is in line with genetic studies [54]. Alternatively, the studies on selectins and integrins are very heterogeneous in terms of trauma severity, therapy, laboratory protocols and timing of sampling. Therefore, standardized prospective studies are mandated to determine the exact clinical course of activation markers after trauma. Furthermore, cell surface expression levels on the systemic neutrophil pool may be affected by shifts of cells into and from other compartments (lungs, bone marrow, lymphoid organs), marginated pool as well as by recovery of expression over time and cell death/apoptosis. The impact of these processes has been studied in trauma and non-trauma experimental studies in the past. Clinical correlation of these findings and future translational studies may boost insights in these processes.

Additionally, *Fcγ-*Receptor CD16 showed a homogeneous decreasing trend after trauma in all available studies. However, long term kinetics are relatively unexplored [18–23]. CD32 on the other hand tends to decrease in the majority of studies, whereas in three studies no effect was seen [21, 27, 28]. Differences in trauma severity or timing between the studies may explain the variability between the studies. CD64, showed a uniform increase of cell surface expression after trauma [13, 29–31].

Interestingly, less frequently studied receptors also showed homogeneous effects after trauma. CD88, decreased after trauma in all 6 studies [20–24, 45]. CD182, decreased in all trauma studies following insult [20, 22, 46, 47]. Several markers have only been investigated by a single study, CD13 and CD46 dropped after trauma [45, 48], CD55 increased following insult [45]. Increased CD59 cell surface expression levels are associated with the development of sepsis [45]. More studies are needed to identify the role of these receptors in the post-traumatic neutrophil response after trauma. Standardized prospective clinical studies may help identifying their potential for post-traumatic immune monitoring.

The predictive value of systemic neutrophil monitoring may further increase by combining several markers. Besides, in-vitro stimulation assays may provide important additional information. Future prospective studies are required to test antibody combinations and their potential to predict outcome after trauma. Technological developments, such as multichannel flowcytometry and automatized flow have enabled such analysis these days and opens the door for point-of-care measurements in trauma bay. The aim of the current study was to address the missing link by identifying feasible antibodies.

## Conclusion

The following systemic literature review on neutrophil receptor expression alterations after trauma resulted in the identification of a unique multi-parameter neutrophil receptor expression pattern, combining well know traditional neutrophil markers for neutrophil activation and maturation (CD11b, CD11a, CD62L, CD16, CD64) as well as markers in which biological relevance in post-traumatic acute systemic inflammation is relatively unexplored (CD88, CD182). Additionally, some novel markers with post-traumatic monitoring potential have been identified (CD13, CD46, CD55, CD59). Combining these markers from literature resulted in the identification of a unique post-traumatic neutrophil signature and may form the basis for immunomonitoring after trauma, especially.

## Electronic Supplementary Material

Below is the link to the electronic supplementary material.

Supplementary Material 1

## Data Availability

No datasets were generated or analysed during the current study.

## References

[1] Keel M, Trentz O. Pathophysiology of polytrauma. Injury. 2005;36:691–709. 15910820 10.1016/j.injury.2004.12.037

[2] Huber-Lang M, Lambris JD, Ward PA. Innate immune responses to trauma. Nat Immunol. 2018;19:327–41. 29507356 10.1038/s41590-018-0064-8 PMC6027646

[3] Pape H-C, Moore EE, McKinley T, Sauaia A. Pathophysiology in patients with polytrauma. Injury. 2022;53:2400–12. 35577600 10.1016/j.injury.2022.04.009

[4] van Breugel JMM, Niemeyer MJS, Houwert RM, Groenwold RHH, Leenen LPH, van Wessem KJP. Global changes in mortality rates in polytrauma patients admitted to the ICU—a systematic review. World J Emerg Surg. 2020;15:55. 32998744 10.1186/s13017-020-00330-3 PMC7526208

[5] Balogh ZJ. Polytrauma: it is a disease. Injury. 2022;53:1727–9. 35643732 10.1016/j.injury.2022.05.001

[6] Tisherman SA, Schmicker RH, Brasel KJ, Bulger EM, Kerby JD, Minei JP, et al. Detailed description of all deaths in both the shock and traumatic brain injury hypertonic saline trials of the resuscitation outcomes consortium. Ann Surg. 2015;261:586–90. 25072443 10.1097/SLA.0000000000000837 PMC4309746

[7] Moore FA, Sauaia A, Moore EE, Haenel JB, Burch JM, Lezotte DC. Postinjury multiple organ failure: A bimodal phenomenon. J Trauma Acute Care Surg. 1996;40:501. 10.1097/00005373-199604000-00001 8614027

[8] Volpin G, Pfeifer R, Saveski J, Hasani I, Cohen M, Pape H-C. Damage control orthopaedics in polytraumatized patients- current concepts. J Clin Orthop Trauma. 2021;12:72–82. 33716431 10.1016/j.jcot.2020.10.018 PMC7920204

[9] Mortaz E, Zadian SS, Shahir M, Folkerts G, Garssen J, Mumby S et al. Does Neutrophil Phenotype Predict the Survival of Trauma Patients? Front Immunol [Internet]. 2019 [cited 2025 Apr 19];10. Available from: https://www.frontiersin.orghttps://www.frontiersin.org/journals/immunology/articles/10.3389/fimmu.2019.02122/full 10.3389/fimmu.2019.02122 PMC6743367 31552051

[10] Teuben MPJ, Pfeifer R, Horst K, Simon T-P, Heeres M, Kalbas Y, et al. Standardized Porcine unilateral femoral nailing is associated with changes in PMN activation status, rather than aberrant systemic PMN prevalence. Eur J Trauma Emerg Surg. 2022;48:1601–11. 34114052 10.1007/s00068-021-01703-2 PMC9192391

[11] Teuben M, Heeres M, Blokhuis T, Hollman A, Vrisekoop N, Tan E, et al. Instant intra-operative neutropenia despite the emergence of banded (CD16dim/CD62Lbright) neutrophils in peripheral blood - An observational study during extensive trauma-surgery in pigs. Injury. 2021;52:426–33. 33208273 10.1016/j.injury.2020.11.018

[12] Hesselink L, Spijkerman R, van Wessem KJP, Koenderman L, Leenen LPH, Huber-Lang M, et al. Neutrophil heterogeneity and its role in infectious complications after severe trauma. World J Emerg Surg. 2019;14:24. 31164913 10.1186/s13017-019-0244-3 PMC6542247

[13] Briggs GD, Lemmert K, Lott NJ, de Malmanche T, Balogh ZJ. Biomarkers to guide the timing of surgery: neutrophil and monocyte L-Selectin predict postoperative Sepsis in orthopaedic trauma patients. J Clin Med. 2021;10:2207. 34065206 10.3390/jcm10102207 PMC8160833

[14] de Fraiture EJ, Bongers SH, Jukema BN, Koenderman L, Vrisekoop N, van Wessem KJP, et al. Visualization of the inflammatory response to injury by neutrophil phenotype categories. Eur J Trauma Emerg Surg. 2023;49:1023–34. 36348032 10.1007/s00068-022-02134-3 PMC10175373

[15] Spijkerman R, Hesselink L, Hellebrekers P, Vrisekoop N, Hietbrink F, Leenen LPH, et al. Automated flow cytometry enables high performance point-of-care analysis of leukocyte phenotypes. J Immunol Methods. 2019;474:112646. 31419409 10.1016/j.jim.2019.112646

[16] Spijkerman R, Hesselink L, Bongers S, van Wessem KJP, Vrisekoop N, Hietbrink F, et al. Point-of-Care analysis of neutrophil phenotypes: A first step toward Immuno-Based precision medicine in the trauma ICU. Crit Care Explorations. 2020;2:e0158. 10.1097/CCE.0000000000000158 PMC7371075 32766555

[17] Wang J. Neutrophils in tissue injury and repair. Cell Tissue Res. 2018;371:531–9. 29383445 10.1007/s00441-017-2785-7 PMC5820392

[18] Johansson J, Sjögren F, Bodelsson M, Sjöberg F. Dynamics of leukocyte receptors after severe burns: an exploratory study. Burns. 2011;37:227–33. 20934812 10.1016/j.burns.2010.08.015

[19] Shih HC, Su CH, Lee CH. Alternations of surface antigens on leukocytes after severe injury: correlation with infectious complications. Intensive Care Med. 1998;24:152–6. 9539073 10.1007/s001340050537

[20] Visser T, Hietbrink F, Groeneveld KM, Koenderman L, Leenen LPH. Isolated blunt chest injury leads to transient activation of Circulating neutrophils. Eur J Trauma Emerg Surg. 2011;37:177–84. 21837259 10.1007/s00068-010-0041-x PMC3150797

[21] Baëhl S, Garneau H, Le Page A, Lorrain D, Viens I, Svotelis A, et al. Altered neutrophil functions in elderly patients during a 6-month follow-up period after a hip fracture. Exp Gerontol. 2015;65:58–68. 25797136 10.1016/j.exger.2015.03.009

[22] Hazeldine J, Naumann DN, Toman E, Davies D, Bishop JRB, Su Z, et al. Prehospital immune responses and development of multiple organ dysfunction syndrome following traumatic injury: A prospective cohort study. PLoS Med. 2017;14:e1002338. 28719602 10.1371/journal.pmed.1002338 PMC5515405

[23] Weiss M, Gueldue Z, Georgieff M, Gebhard F, Huber-Lang M, Schneider M. Polymorphonuclear cell surface expression patterns differ in inflammatory and infectious stages in polytraumatized and septic shock patients. Crit Care. 2014;18:P228.

[24] Hietbrink F, Koenderman L, Althuizen M, Pillay J, Kamp V, Leenen LPH. Kinetics of the innate immune response after trauma: implications for the development of late onset sepsis. Shock. 2013;40:21–7. 23603769 10.1097/SHK.0b013e318295a40a

[25] Hietbrink F, Koenderman L, Leenen LP. Intramedullary nailing of the femur and the systemic activation of monocytes and neutrophils. World J Emerg Surg. 2011;6:34. 22040874 10.1186/1749-7922-6-34 PMC3216875

[26] Groeneveld KM, Koenderman L, Warren BL, Jol S, Leenen LPH, Hietbrink F. Early decreased neutrophil responsiveness is related to late onset sepsis in multitrauma patients: an international cohort study. PLoS ONE. 2017;12:e0180145. 28665985 10.1371/journal.pone.0180145 PMC5493351

[27] Hietbrink F, Koenderman L, van Wessem KJP, Leenen LPH. The impact of intramedullary nailing of tibia fractures on the innate immune system. Shock. 2015;44:209. 26009818 10.1097/SHK.0000000000000405

[28] Hietbrink F, Koenderman L, Althuizen M, Leenen LPH. Modulation of the innate immune response after trauma visualised by a change in functional PMN phenotype. Injury. 2009;40:851–5. 19339006 10.1016/j.injury.2008.11.002

[29] Raikwar A, Singh A, Verma V, Mehdi AA, Kushwaha NS, Kushwaha R. Analysis of risk factors and association of cluster of differentiation (CD) markers with conventional markers in delayed fracture related infection for closed fracture. Cureus. 2021;13:e20124. 35003964 10.7759/cureus.20124 PMC8726508

[30] Wang J, Wen D, Zhong H, Gan L, Du J, Zhang H, et al. [Dynamic changes of cellular immune function in trauma patients and its relationship with prognosis]. Zhonghua Wei Zhong Bing Ji Jiu Yi Xue. 2021;33:223–8. 33729144 10.3760/cma.j.cn121430-20200902-00604

[31] Wu Y, Mao Q, Hong J, Hu Z, Li X, Gong Z. Serum PCT, CRP and CD64 levels in predicting early infection after internal fixation of limb fractures. Chin J Clin Infect Dis. 2023;278–83.

[32] Ley K, Laudanna C, Cybulsky MI, Nourshargh S. Getting to the site of inflammation: the leukocyte adhesion cascade updated. Nat Rev Immunol. 2007;7:678–89. 17717539 10.1038/nri2156

[33] Maekawa K, Futami S, Nishida M, Terada T, Inagawa H, Suzuki S, et al. Effects of trauma and Sepsis on soluble L-Selectin and cell surface expression of L-Selectin and CD11b. J Trauma Acute Care Surg. 1998;44:460. 10.1097/00005373-199803000-00007 9529172

[34] Agudelo JF, Flierl MA, Smith WR, Moore EE, Williams AE, Eckels PC, et al. Influence of preoperative 7.5% hypertonic saline on neutrophil activation after reamed intramedullary nailing of femur shaft fractures: A prospective randomized pilot study. J Orthop Trauma. 2012;26:86. 21904224 10.1097/BOT.0b013e31821cfd2a

[35] Nijdam TMP, Jukema BN, de Fraiture EJ, Spijkerman R, Schuijt HJ, Spoelder M, et al. Identification of neutrophil phenotype categories in geriatric hip fracture patients aids in personalized medicine. OTA Int. 2024;7:e291. 10.1097/OI9.0000000000000291 PMC10750458 38152436

[36] Mommsen P, Barkhausen T, Hildebrand F, Zeckey C, Krettek C, van Griensven M. Regulation of L-selectin expression by trauma-relevant cytokines. Pathol - Res Pract. 2011;207:142–7. 21237580 10.1016/j.prp.2010.12.003

[37] Cocks RA, Chan TYF, Rainer TH. Leukocyte L-Selectin is Up-Regulated after mechanical trauma in adults. J Trauma Acute Care Surg. 1998;45:1. 10.1097/00005373-199807000-00001 9680003

[38] Vestweber D. How leukocytes cross the vascular endothelium. Nat Rev Immunol. 2015;15:692–704. 26471775 10.1038/nri3908

[39] Hietbrink F, Oudijk E-J, Braams R, Koenderman L, Leenen L. Aberrant regulation of polymorphonuclear phagocyte responsiveness in multitrauma patients. Shock. 2006;26:558–64. 17117129 10.1097/01.shk.0000233196.40989.78

[40] Bhatia RK, Pallister I, Dent C, Jones SA, Topley N. Enhanced neutrophil migratory activity following major blunt trauma. Injury. 2005;36:956–62. 15998513 10.1016/j.injury.2005.03.009

[41] Scannell G, Waxman K, Vaziri ND, Zhang J, Kaupke CJ, Jalali M, et al. Effects of trauma on leukocyte intercellular adhesion Molecule-1, CD11b, and CD18 expressions. J Trauma Acute Care Surg. 1995;39:641. 10.1097/00005373-199510000-00004 7473947

[42] Brom J, Köller M, Schlüter B, Müller-Lange P, Steinau HU, König W. Expression of the adhesion molecule CD11b and polymerization of actin by polymorphonuclear granulocytes of patients endangered by sepsis. Burns. 1995;21:427–31. 8554683 10.1016/0305-4179(95)00011-y

[43] Bhatia R, Dent C, Topley N, Pallister I. Neutrophil priming for elastase release in adult blunt trauma patients. J Trauma Acute Care Surg. 2006;60:590. 10.1097/01.ta.0000205614.51885.ff 16531859

[44] Botha AJ, Moore FA, Moore EE, Kim FJ, Banerjee A, Peterson VM. Postinjury neutrophil priming and activation: an early vulnerable window. Surgery. 1995;118:358–64. discussion 364–365. 7638753 10.1016/s0039-6060(05)80345-9

[45] Amara U, Kalbitz M, Perl M, Flierl MA, Rittirsch D, Weiss M, et al. EARLY EXPRESSION CHANGES OF COMPLEMENT REGULATORY PROTEINS AND C5a RECEPTOR (CD88) ON LEUKOCYTES AFTER MULTIPLE INJURY IN HUMANS. Shock. 2010;33:568. 19864971 10.1097/SHK.0b013e3181c799d4

[46] Tarlowe MH, Duffy A, Kannan KB, Itagaki K, Lavery RF, Livingston DH, et al. Prospective study of neutrophil chemokine responses in trauma patients at risk for pneumonia. Am J Respir Crit Care Med. 2005;171:753–9. 15618463 10.1164/rccm.200307-917OC

[47] Quaid GA, Cave C, Robinson C, Williams MA, Solomkin JS. Preferential loss of CXCR-2 receptor expression and function in patients who have undergone trauma. Arch Surg. 1999;134:1367–72. 10593336 10.1001/archsurg.134.12.1367

[48] HUSCHAK G, ZUR NIEDEN K, STUTTMANN R. Changes in monocytic expression of aminopeptidase N/CD13 after major trauma. Clin Exp Immunol. 2003;134:491–6. 14632756 10.1111/j.1365-2249.2003.02302.x PMC1808881

[49] Drifte G, Dunn-Siegrist I, Tissières P, Pugin J. Innate immune functions of immature neutrophils in patients with Sepsis and severe systemic inflammatory response syndrome**. Crit Care Med. 2013;41:820. 23348516 10.1097/CCM.0b013e318274647d

[50] Orr Y, Taylor JM, Bannon PG, Geczy C, Kritharides L. Circulating CD10–/CD16low neutrophils provide a quantitative index of active bone marrow neutrophil release. Br J Haematol. 2005;131:508–19. 16281943 10.1111/j.1365-2141.2005.05794.x

[51] Bhatia R, Dent C, Topley N, Pallister I. Modulation of Interleukin-8-Mediated neutrophil migration following major Lower-Limb fracture and operative stabilization. Eur J Trauma. 2005;31:575–82.

[52] Skubitz KM, Campbell KD, Skubitz APN. CD66a, CD66b, CD66c, and CD66d each independently stimulate neutrophils. J Leukoc Biol. 1996;60:106–17. 8699114 10.1002/jlb.60.1.106

[53] Botha AJ, Moore FA, Moore EE, Peterson VM, Goode AW. Base deficit after major trauma directly relates to neutrophil CD11b expression: a proposed mechanism of shock-induced organ injury. Intensive Care Med. 1997;23:504–9. 9201521 10.1007/s001340050365

[54] Xiao W, Mindrinos MN, Seok J, Cuschieri J, Cuenca AG, Gao H, et al. A genomic storm in critically injured humans. J Exp Med. 2011;208:2581–90. 22110166 10.1084/jem.20111354 PMC3244029

