# Supplementary material for: Neutrophil cell surface receptor dynamics following trauma: a systematic review
Source: Eur J Trauma Emerg Surg. 2025 Aug 11;51(1):270. doi: 10.1007/s00068-025-02937-0 (PMC12339592; doi:10.1007/s00068-025-02937-0)
Supplement: Supplementary file 1 — Supplementary Material 1 [file 68_2025_2937_MOESM1_ESM.docx]

Supplementary File 1

Search Terms of the systematic review

**MEDLINE**

"fractures, bone"[MeSH Terms] OR "Multiple Trauma"[MeSH Terms] OR "fractur*"[Title/Abstract] OR "monotraum*"[Title/Abstract] OR "severe trauma"[Title/Abstract] OR "severely traum*"[Title/Abstract] OR "Multiple Trauma"[Title/Abstract] OR "polytrauma"[Title/Abstract] OR "polytraum*"[Title/Abstract]

AND

"Neutrophils"[Mesh] OR “neutrophi*”[Title/Abstract] OR "Flow Cytometry"[Mesh] OR “cytomet*”[Title/Abstract]

AND

"Receptors, Cell Surface"[Mesh] OR "Integrins"[Mesh] OR “receptor”[Title/Abstract] OR “CD”[Title/Abstract] OR “express*”[Title/Abstract]

AND

("1995/01/01"[Date - Publication]: "2023/12/31"[Date - Publication])

**EMBASE**

'multiple trauma'/exp OR 'fracture'/exp OR 'severe trauma':ab,ti OR 'severely trauma*':ab,ti OR 'fractur*':ab,ti OR 'monotrau*':ab,ti OR 'multiple trauma*':ab,ti OR 'polytrauma':ab,ti OR 'polytraum*':ab,ti

AND

'neutrophil'/exp OR 'flow cytometry'/exp OR 'neutrophi*':ab,ti OR 'cytomet*':ab,ti

AND

'cell surface receptor'/exp OR 'membrane receptor'/exp OR 'integrin'/exp OR 'receptor*':ab,ti OR 'CD':ab,ti OR 'express*':ab,ti

AND

[1995-2023]/py
